# The multimodal imaging features and outcomes of multifocal choroiditis/punctate inner choroidopathy lesion with multiple evanescent white dot syndrome-like features: a retrospective study

**DOI:** 10.1186/s12886-023-03277-6

**Published:** 2024-01-02

**Authors:** Chunli Chen, Yizhe Cheng, Zhihan Zhang, Yang Zhang, Simeng Hou, Ge Wang, Xiaoyan Peng

**Affiliations:** 1 Department of Ophthalmology, Beijing Tongren Hospital, Capital Medical University, Beijing, China; 2 Beijing Ophthalmology and Visual Science Key Laboratory, Beijing, China; 3 State Key Laboratory of Ophthalmology, Zhongshan Ophthalmic Center, Sun Yat-sen University, Guangzhou, China; 4 Beijing Institute of Ophthalmology, Beijing, China

**Keywords:** Multifocal choroiditis/punctate inner choroidopathy, Multiple evanescent white dot syndrome, Multimodal imaging, Focal choroidal excavation, Choroidal neovascularization

## Abstract

**Background:**

Multiple evanescent white dot syndrome (MEWDS)-like features is a rare condition triggered by a macular disease or iatrogenic injury, exhibiting MEWDS changes in the fundus. This study aims to describe the multimodal imaging features and outcomes of multifocal choroiditis/punctate inner choroidopathy (MFC/PIC) lesions with MEWDS-like features.

**Methods:**

Six cases were studied retrospectively. All cases were given regional and oral corticosteroids.

**Results:**

All cases showed an isolated juxtafoveal yellowish-white MFC/PIC lesion with disruption of RPE-Bruch’s membrane-choriocapillaris complex (RPE-BM-CC), subretinal hyperreflective materials and choroidal thickening on optical coherence tomography. Two weeks after presentation, the grayish-white dots disappeared spontaneously and the corticosteroids were given. After four weeks, the ellipsoid zone (EZ) around the lesion and hyper-autofluorescence resolved. After 13 weeks, five cases showed shrinkage of the juxtafoveal lesion and restoration of foveal EZ. After six months, the juxtafoveal lesion became pigmented. Only one case developed type 2 choroidal neovascularization.

**Conclusions:**

The clinical course of MEWDS-like manifestations is still evanescent in our cases. The yellowish-white juxtafoveal MFC/PIC lesions with disruption of RPE-BM-CC and choroidal thickening showed a well-controlled prognosis after corticosteroid treatment.

## Summary statement

This study is a retrospective case series to describe the multimodal imaging features and outcomes of multifocal choroiditis/punctate inner choroidopathy lesion with multiple evanescent white dot syndrome-like features.

## Introduction

Multiple evanescent white dot syndrome (MEWDS) is a kind of multifocal chorioretinal disorder, first reported by Jampol in 1984, [1] typically occurring in young myopic women with symptoms of moderate to severe vision loss, photopsia, and enlargement of the physiologic scotoma [2–4]. The etiology of MEWDS remains unknown. The elevation of virus-related IgM and IgG in MEWDS cases suggests viral infection that possibly triggered the occurrence of MEWDS [5, 6]. The diagnosis mainly relies on medical history, typical fundus changes, and ancillary multimodal imaging (MMI) examinations [5]. Clinically, MEWDS is characterized by multifocal grayish-white spots in the deep retina of posterior pole and periphery with foveal granularity or optic disc edema in the acute phase. Anterior chamber and vitreous inflammation can also be noted in some cases [7].

MEWDS often shows a self-limiting course with a duration of 6–8 weeks and a good prognosis with complete restoration of grayish spots and no disruption of RPE-Bruch’s membrane-choriocapillaris complex (RPE-BM-CC). Therefore, regional or systemic corticosteroids therapy has been not regularly used [2]. However, in an era of MMI, many reported cases show MEWDS-like reaction associated with a former or concurrent retinal disease [8, 9]. Yannuzzi et al. [10] have recently proposed an entity termed as ‘Secondary MEWDS’ that may be triggered by a macular disease or iatrogenic injury, distinguishing from the typical MEWDS described by Jampol et al. [1] Despite there are varied novel terms identifying a spectrum of white dot syndrome, [10–13] there still have been many overlaps in this spectrum hardly to be categorized.

Although the aforementioned classic presentation with a self-limiting course of primary MEWDS, focal choroidal excavation (FCE) [14–16] and choroidal neovascularization (CNV) [8, 17–22] are incidental complications in cases with inflammatory chorioretinal lesions. In this article, we summarize the MMI features of isolated juxtafoveal yellowish-white multifocal choroiditis/punctate inner choroidopathy (MFC/PIC) lesions with MEWDS-like features and report the outcomes after corticosteroid treatment.

## Materials and methods

This was a retrospective case study that abided by the Declaration of Helsinki, and informed consent was waived by the ethics committee of Beijing Tongren Hospital due to the study’s retrospective nature. The diagnostic criteria of MEWDS or MEWDS-like features referenced the published criteria [5] as follows: (1) multifocal grayish-white spots with foveal granularity on ophthalmoscope or color fundus photograph (CFP); (2) diffuse or focal disruption of ellipsoid zone (EZ) and hyperreflective lesions overlying RPE on optical coherence tomography (OCT); (3) scattered or fused hyper-autofluorescence (AF) in the posterior pole and around optic disc on fundus autofluorescence (FAF); (4) “wreath-like” hyperfluorescent lesions on fundus fluorescein angiography (FFA) and hypofluorescent dots on indocyanine green angiography (ICGA); (5) The aforementioned fundus features affiliated to MEWDS could ultimately resolve during the follow-up period. Apart from typical features of MEWDS, the cases that simultaneously showed a juxtafoveal yellowish-white MFC/PIC lesion with disruption of RPE-BM-CC and choroidal thickening on structural OCT were selected. Exclusion criteria: cases with other white spot syndromes, especially the entity typically presenting multifocal chorioretinal lesions, were excluded by MMI. All patients underwent examination of the immune-related and infectious blood test, TORCHES infection test (toxoplasmosis, rubella, cytomegalovirus, and herpes simplex virus), antinuclear antibodies, tuberculosis, and chest computed tomography (CT). Data collection included age, gender, medical history, best corrected visual acuity (BCVA) (Snellen), refractive status, slit lamp examination, and ophthalmoscopy. All six patients underwent MMI examinations including CFP (CR-2 AF automatic no-dilatation fundus camera, CANON, Japan), FAF, and OCT (Heidelberg SPETRALIS HRA + OCT, Heidelberg, Germany) and swept source-OCT (SS-OCT) (VG100; SVision Imaging, Ltd., Luoyang, China). In addition, five cases had FFA, and three cases had ICGA (Heidelberg SPETRALIS HRA + OCT, Heidelberg, Germany) and angio-optical coherence tomography (OCTA) examination (RTVue Version 4.0, Optovue, USA or VG100; SVision Imaging, Ltd., Luoyang, China). The choroidal thickness under the yellowish-white lesions was measured by the OCT line-scan mode. The subfoveal choroidal thickness was measured on structural OCT lines scans using the caliper function (Heidelberg Eye Explorer V 6.3.3.0; Heidelberg Engineering). Data were displayed as mean ± standard deviation. All the time points mentioned in our study were relative to the time of the initial presentation.

## Result

### Demographic and baseline characteristics

There were six eyes of six enrolled patients (5 female, 1 male) with a median age of 33.3 ± 10.43 years (range 17–50 years). Four cases had anterior chamber cells and vitreous inflammatory cells. The median follow-up time was 18.0 ± 16.53 months (range: 6–56 months). Five cases had spherical equivalent refractive error ranging from − 2.0 diopters to -8.0 diopters, including 1 case with high myopia. All patients were negative for infectious and immune-related blood tests. All patients were treated with at least one retrobulbar injection of triamcinolone acetonide (TA) 20 mg at presentation. After the exclusion of active systematic infectious disease, oral prednisone was initiated with a dose of 1.0 mg/Kg, tapering gradually.

### CFP changes during the follow-up period

**Fig. 1:**
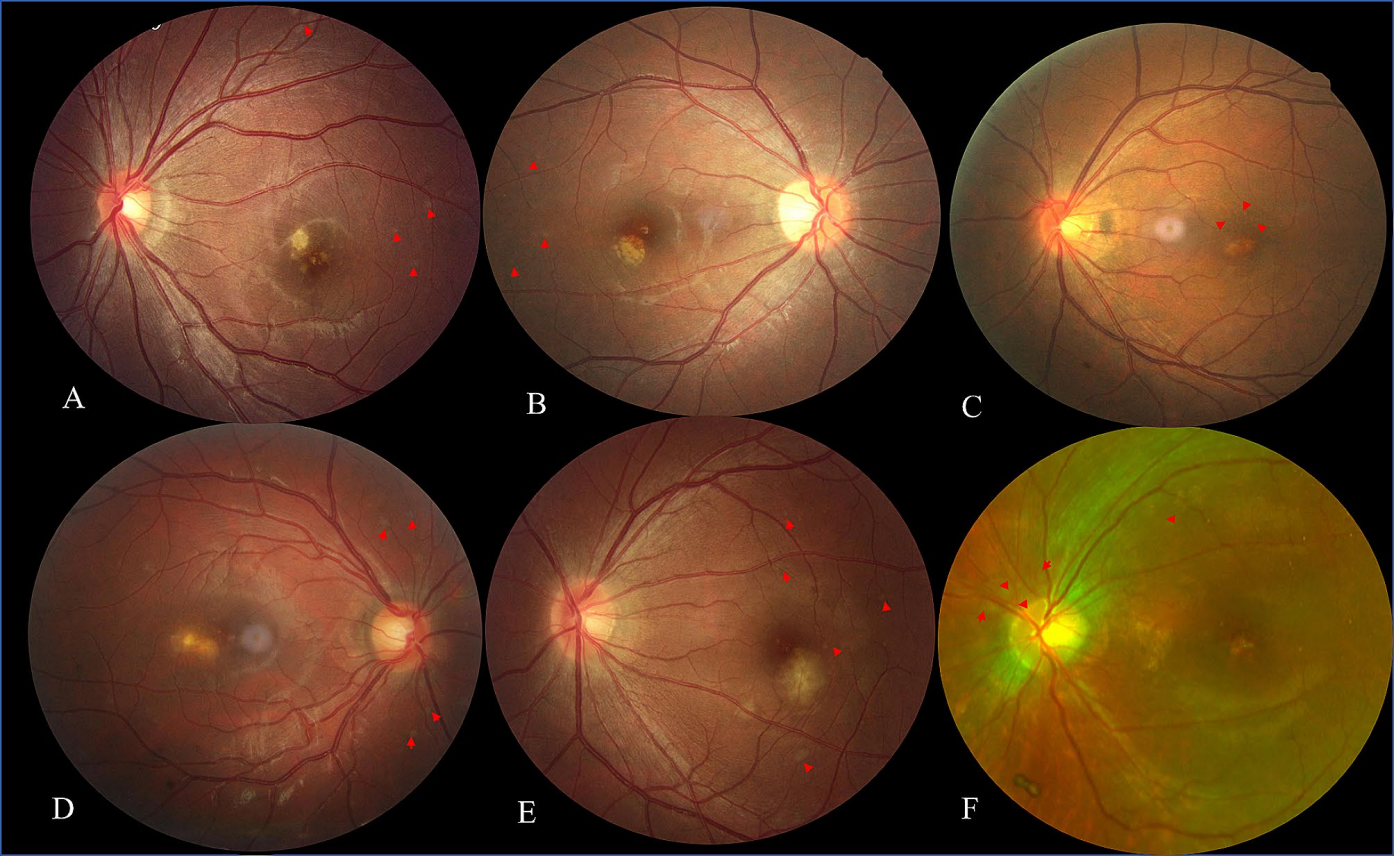
Yellowish-white juxtafoveal lesion in six cases (**A-F**) of different shapes and sizes. (Red arrows show the white dots.)

At presentation, the CFP of six patients showed multifocal grayish-white dots in the deep retina of the posterior pole, with foveal granularity and an isolated yellowish-white lesion in the juxtafovea (Fig. 1). Two weeks later, the grayish-white dots in the posterior pole largely disappeared without any treatment. The corticosteroids were given after referral to our clinic. After seven weeks, foveal granularity reduced or even disappeared. After 13 weeks, the yellowish-white juxtafoveal lesion of five cases shrank and became better defined. After six months, the yellowish-white lesion became pigmented. (Table 1)

**Table 1:** The recovery situation of gray-white lesions, granular changes and yellow-white MFC/PIC lesions

Case	Gender	Age	Myopia Level	Gray-white dots		Foveal granularity		MFC/PIC lesion			FAF	
				Recovery time	Outcome	Recovery time	Outcome	location	Recovery time	Outcome	Recovery time	Outcome
1	Female	17	Moderate	1w + 3d	Few in temporal macula	4w	Disappeared	Juxtafovea	8w	Stablized	6w	Disappeared
2	Female	36	Moderate	3w	Disappeared	6w	Disappeared	Juxtafovea	8w	Stablized	3w	Disappeared
3	Male	39	Moderate	1w + 3d	Disappeared	12w	Relieved	Juxtafovea	16w	Stablized	4w	Disappeared
4	Female	25	Moderate	3w	Disappeared	8w	Disappeared	Juxtafovea	16w	Stablized	3w	Disappeared
5	Female	50	Mild	4w	Disappeared	6w	Disappeared	Juxtafovea	10w	Stablized	4w	Disappeared
6	Female	33	High	2w	Disappeared	4w	Disappeared	Juxtafovea	10w	Stablized	4w	Disappeared

### OCT changes during the follow-up period

**Fig. 2:**
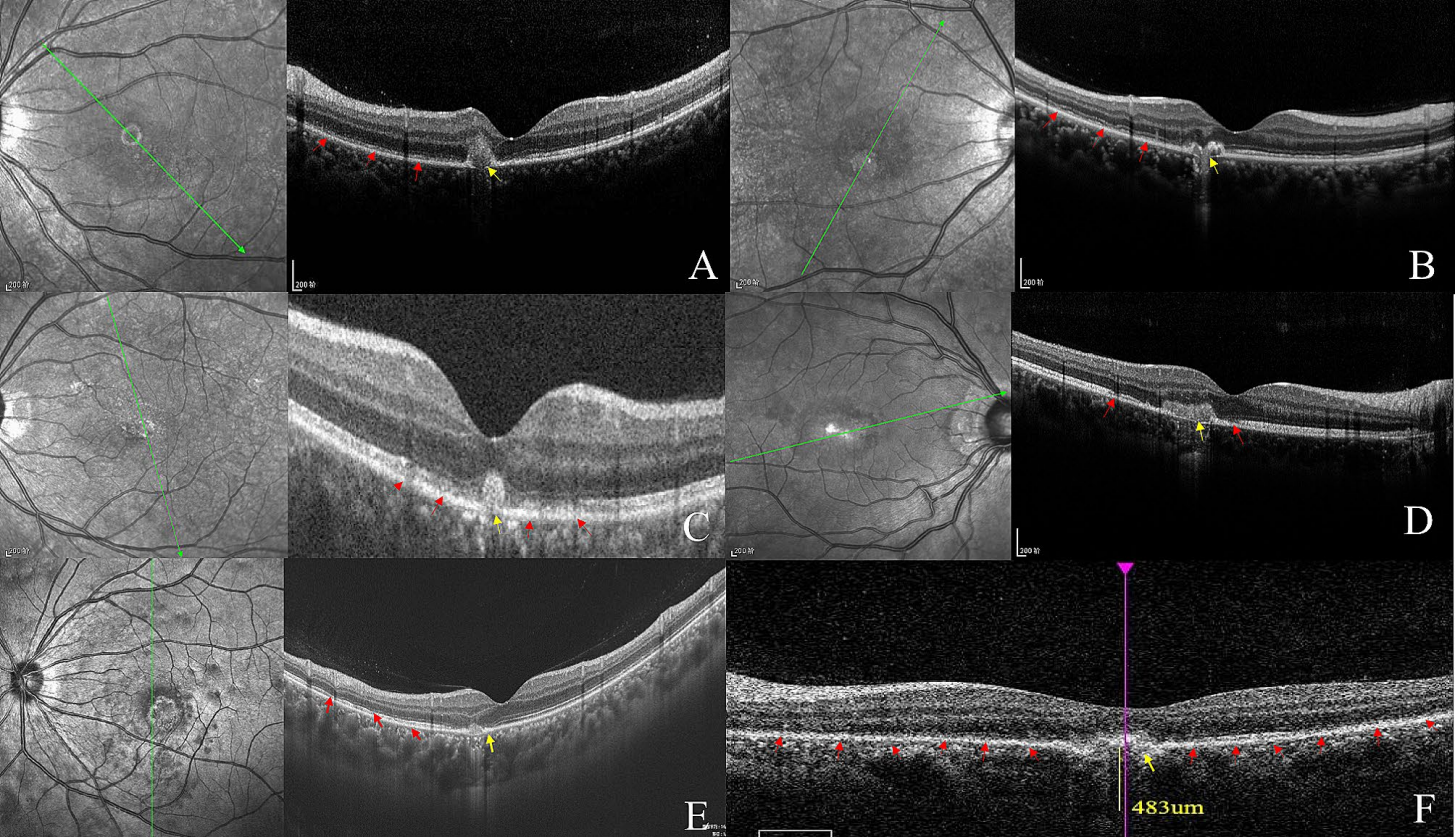
Disruption of the ellipsoid zone around the lesion and disruption of RPE-BM-CC at the lesion’s site on OCT (**A-F** Case 1–6)

At presentation, all cases presented with inhomogeneous subretinal hyperreflective materials (SHRM) and disruption of RPE-BM-CC at the yellowish-white lesion’s site on OCT (Fig. 2). OCT also showed disruption of the ellipsoid zone (EZ) associated with hypertrophy of the underlying retinal pigment epithelium (RPE) around the lesion. After four weeks, the hypertrophy of the RPE was restored. The perifoveal EZ was restored after 4 weeks and the foveal EZ was restored after 13 weeks. The SHRM at the yellowish’s site showed an obvious decrease or restoration after 17 weeks. The disruption of RPE-BM-CC was mainly located in juxtafovea. (Table 2). Of all the cases, the subfoveal choroidal thickness of the affected eyes was thicker (range 274 to 483 μm) than that of the fellow eyes. As for the affected eyes, the subfoveal choroidal thickness in the active phase was an average of roughly 20 μm thicker than that in the stabilized phase.

**Table 2:** The recovery situation and location of the Elipsode zone and RPE-Bruch’s membrane-choriocapillaris complex lesion on OCT. RPE-BM-CC, RPE-Bruch’s membrane-choriocapillaris complex

OCT												
	Elipsode zone				Hyperreflective material				RPE-BM complex		Vitreous opacities	
Case	Fovea	Recovery time	Peripheral area	Recovery time	Fovea	Recovery time	Peripheral area	Recovery time	Location	Disruption	Recovery time	Outcome
1	nasal disruption	18w	Recovery	8w	disappeared	18w	disappeared	6w	Super-nasal parafovea	+	8w	Recovery
2	Recovery	10w	Recovery	4w	disappeared	18w	disappeared	4w	Infer-temporal parafovea	+	3w	Recovery
3	Recovery	12w	Recovery	3w	disappeared	14w	disappeared	3w	Subfovea	+	3w	Recovery
4	Temporal disruption	16w	Recovery	2w	stable	20w	disappeared	4w	Infer-temporal parafovea	+	8w	Recovery
5	Temporal disruption	12w	Recovery	4w	stable	18w	disappeared	4w	Super-nasal parafovea	+	3w	Recovery
6	nasal disruption	12w	Recovery	5w	stable	12w	disappeared	4w	Infer-nasal parafovea	+	4w	Recovery

### Other related ocular changes during the follow-up period

Four weeks later, the hyper-AF disappeared on FAF. The inflammatory cells were visible in the vitreous body, which disappeared after 7 weeks. In addition, Type 2 CNV occurred in case 4 when the prednisone was reduced to 10 mg. At the last follow-up visit, five cases had Snellen corrected visual acuity of 20/20, and only one case with CNV had a worse visual acuity of 20/100.

To better describe the clinical history, Case 1 to 4 were selected as representative cases to make case presentations. Case 5 and 6 are respectively similar to Case 1 and 3, so we don’t redundantly expatiate Case 5 and 6.

### Case presentation

#### Case 1

**Fig. 3:**
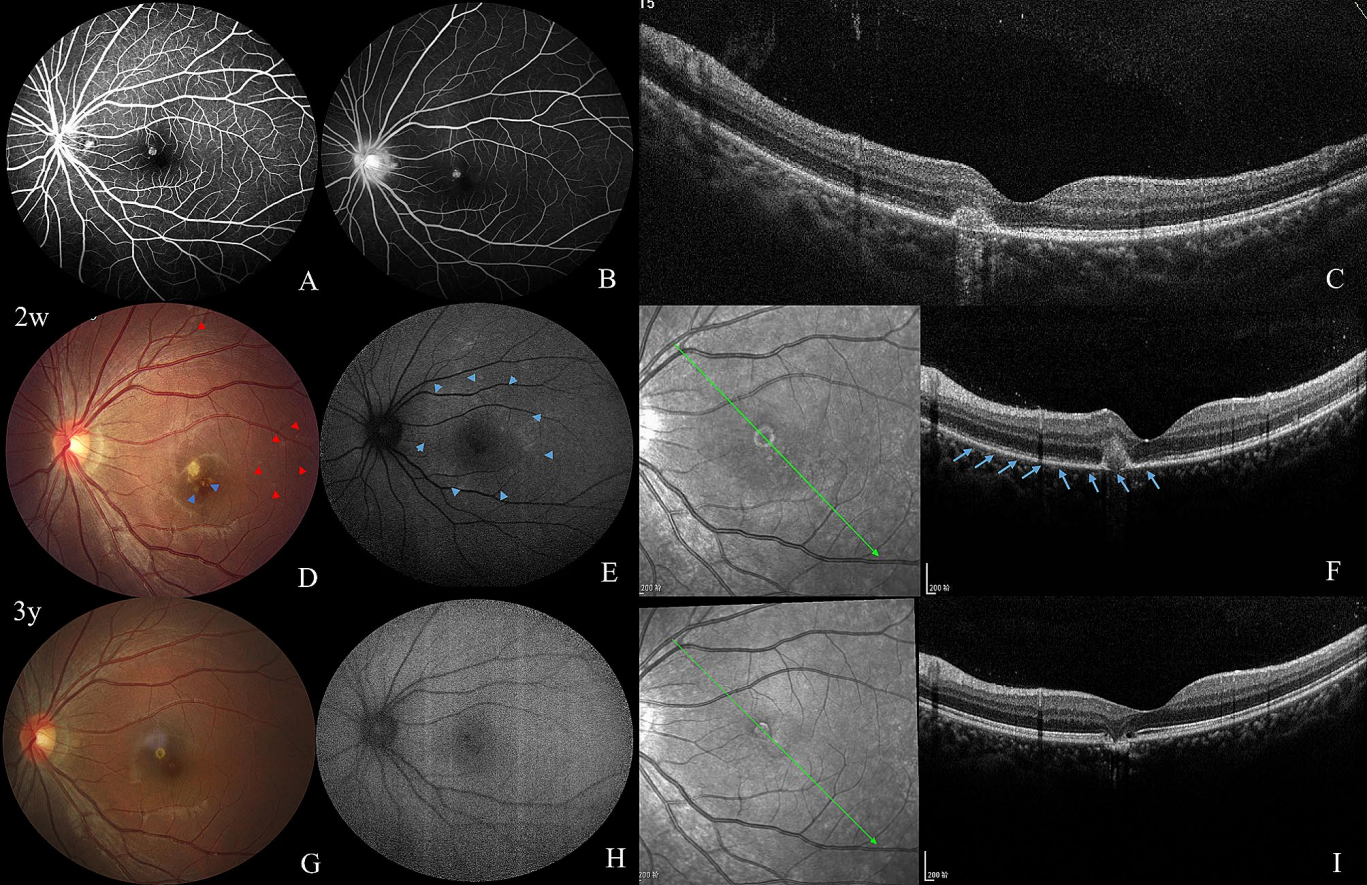
Case 1: The first visit at the local clinic (**A-C**). 2 weeks later visiting our clinic (**D-F**) and a follow-up visit after 3 years (**G-I**). **A**: Early-phase FFA showed hyperfluorescence in a cluster and wreath-like pattern. **B**: Late-phase FFA showed a hot optic disc. **C**: Hyperreflective lesion in the subnasal area of fovea with disruption of the RPE-BM-CC and EZ on OCT. **D**: Diffuse posterior pole grey-white dots (red arrow), subnasal foveal yellowish-white lesion, and foveal granularity (blue arrow). **E**: Scattered hyperautofluorescent dots in the posterior pole on FAF (blue arrow). **F**: Juxtafoveal hyperreflective materials disrupted the RPE and disruption of EZ around the lesion on OCT. **G**: Yellowish-white lesion became better defined and pigmented. **H**: The juxtafoveal lesion showed hypoautofluorescence on FAF. **I**: OCT showed restoration of foveal EZ and RPE-BM-CC and sagged middle layer of the retina, and focal EZ and RPE loss at the lesion’s site

A 17-year-old female presented with central scotoma of the left eye for more than ten days and she visited a local clinic initially. The former medical history showed recurrent low fever for 2 years and her BCVA was oculus unati (OU) 20/20. FFA that was done at the local clinic showed wreath-like hyperfluorescence in the early phase (Fig. 3A), and hot optic disc in the late phase (Fig. 3B). OCT that was done at the local clinic showed a hyperreflective lesion with disruption of RPE-BM-CC and EZ in the parafovea (Fig. 3C). Other medical records at the local clinic were not applicable. Ten days later, she was referred to our clinic. Slit-lamp examination showed that the anterior segment and vitreous cavity were quiet. Funduscopic examination of the left eye showed diffuse grayish-white dots in the posterior pole, as well as a juxtafoveal yellowish-white lesion and foveal granularity (Fig. 3D). The grayish-white dots were scattered hyper-AF in the posterior pole on FAF (Fig. 3E). The juxtafoveal SHRM with disruption of RPE and EZ was noted on OCT (Fig. 3F). These imaging changes corresponded to the diagnosis of MEWDS. The patient was treated with retrobulbar TA injection for three times and oral prednisone for three months. After three years, CFP showed that the yellowish-white lesion became pigmented and smaller than before (Fig. 3G), corresponding to the hypoautofluorescence on FAF (Fig. 3H). OCT showed sagged middle layer of the retina and loss of EZ and RPE at the lesion’s site. Nevertheless, the EZ around the lesion was restored and the Bruch’s membrane was continuous on OCT. (Fig. 3I).

#### Case 2

**Fig. 4:**
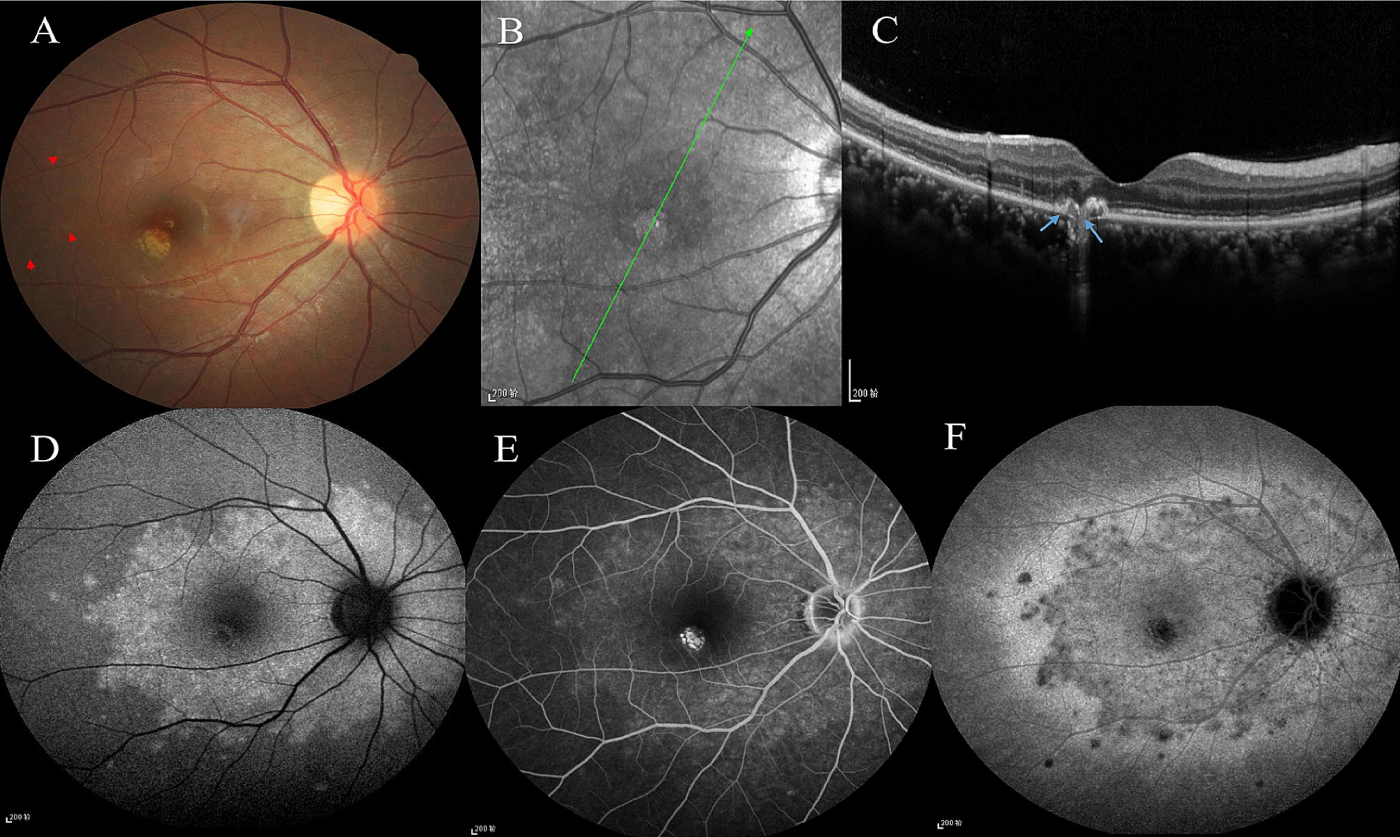
Case 2: **A**: A few grayish-white dots and a yellowish-white lesion in subtemporal juxtafovea on CFP. **B-C**: Segmental disruption of EZ around the lesion, disruption of RPE-BM-CC and SHRM at the lesion’s site on OCT (blue arrows show the disruption of RPE-BM-CC). **D**: Scattered hyperautofluorescent dots and spots in the posterior pole on FAF. **E**: Diffuse and wreath-like hyperfluorescence in the posterior pole on FFA. **F**: Scattered hypofluorescent dots and spots in the posterior pole on ICGA.

A 36-year-old female presented with decreased visual acuity and photopsia in the right eye for two weeks. Previous medical history showed the spherical equivalent refractive error of − 2.75 diopters oculus dexter (OD) and − 2.50 diopters oculus sinister (OS). Her BCVA was OU 20/20. On CFP, several grayish-white dots and a juxtafoveal yellowish-white lesion could be observed in the right eye (Fig. 4A). OCT showed SHRM and disruption of RPE-BM-CC at the yellowish-white lesion’s site, as well as segmental disruption of EZ around the lesion (Fig. 4B&C). FAF showed diffuse hyperautofluorescent dots and spots in the posterior pole (Fig. 4D). FFA showed wreath-like hyperfluorescence in the posterior pole (Fig. 4E). ICGA showed diffuse hypofluorescent dots and spots in the posterior pole (Fig. 4F). The patient was given two retrobulbar injections of TA and oral corticosteroids for three months. The lesion became pigmented and well-defined. The patient was then followed up online with prednisone tapering gradually to complete withdrawal according to the doctor’s guidance.

#### Case 3

**Fig. 5:**
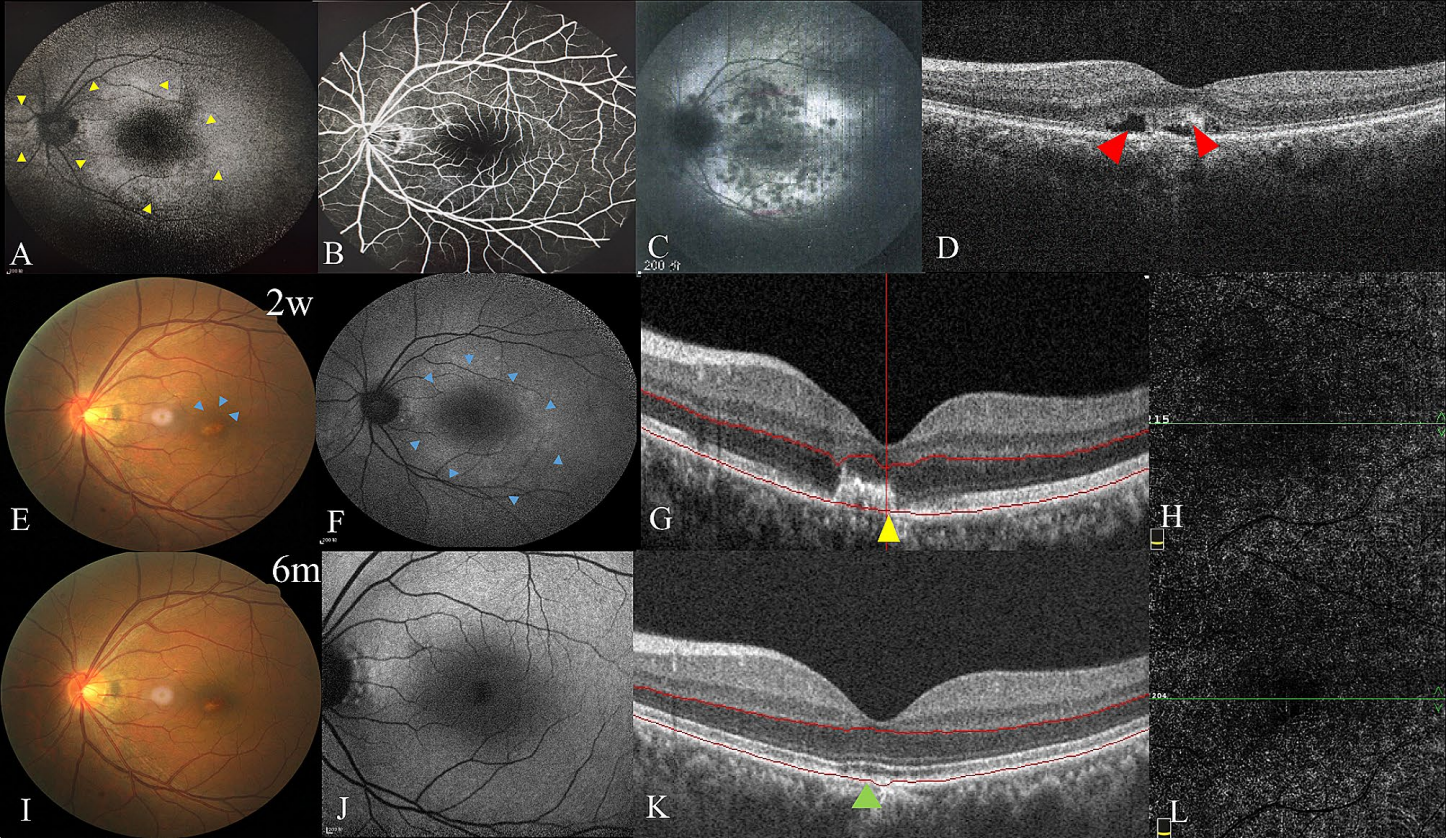
Case 3: First visit at a local clinic (**A-D**). Visit to our clinic two weeks later (**D-H**). Six-month follow-up visit (**I-L**). **A**: Scattered or diffuse hyperautofluorescence (yellow arrow) on FAF. **B**: Early-phase hyperfluorescence in a wreath-like pattern around optic disc on FFA. **C**: Late-phase ICGA showed diffuse and scattered hypofluorescent dots and spots. **D**: OCT showed shallow retinal detachment and hyperreflective materials (red arrow) **E**: CFP showed a yellowish-white lesion in the subnasal fovea (blue arrow). **F**: FAF showed hyperautofluorescent dots and spots (blue arrow). **G**: SHRM disruption of RPE-BM-CC at the lesion’s site and multiple disruptions of EZ around the lesion on OCT. **H**: No neovascularization was found on OCTA. **I**: CFP showed that a yellowish-white lesion in the subnasal fovea became better defined. **J**: FAF showed no abnormalities. **K**: OCT showed no SHRM (green arrow). **L**: No neovascularization on OCTA

A 39-year-old male presented with a fixed black spot and metamorphopsia in his left eye for 18 days. Three days after the presentation, the patient came to the local clinic. He had a cold two weeks ago and allergic rhinitis for more than 20 years. His spherical equivalent refractive error was − 4.5 diopters OD and − 4.0 diopters OS. The OCT, FAF, FFA, and ICGA at presentation were conducted at the local clinic half a month ago. FAF showed hyperautofluorescence around the optic disc and macula (Fig. 5A). Early-phase FFA showed wreath-like hyperfluorescence (Fig. 5B), and late-phase ICGA showed diffuse hypofluorescent dots and spots in the posterior pole (Fig. 5C). These manifestations were in line with the diagnosis of MEWDS. On OCT, shallow retinal detachment and SHRM were noted in the acute phase (Fig. 5D). He was treated with one retrobulbar TA 20 mg injection at the local clinic. Two weeks after the presentation, he was referred to our clinic. The inflammatory cells were noted both in the anterior chamber and vitreous cavity for the left eye, while the right eye was quiet. CFP showed a yellowish-white lesion in the sub-nasal side of the fovea, as well as foveal granularity and grayish-white dots in the posterior pole (Fig. 5E). FAF showed hyperautofluorescent dots (Fig. 5F). OCT showed SHRM and disruption of RPE-BM-CC corresponding to the yellowish-white lesion and multiple disruptions of EZ (Fig. 5G). No neovascularization was found on OCTA (Fig. 5H). The patient was given four-month oral prednisone and a second retrobulbar TA 20 mg injection when the oral prednisone was reduced to 10 mg/day. After six months, CFP showed that the yellowish-white lesion became better defined with pigmentation (Fig. 5I), and no abnormalities on FAF (Fig. 5J). On OCT, the SHRM disappeared and the disruption of EZ and RPE-BM-CC was restored (Fig. 5K). There was still no neovascularization flow detected on OCTA (Fig. 5L).

#### Case 4

**Fig. 6:**
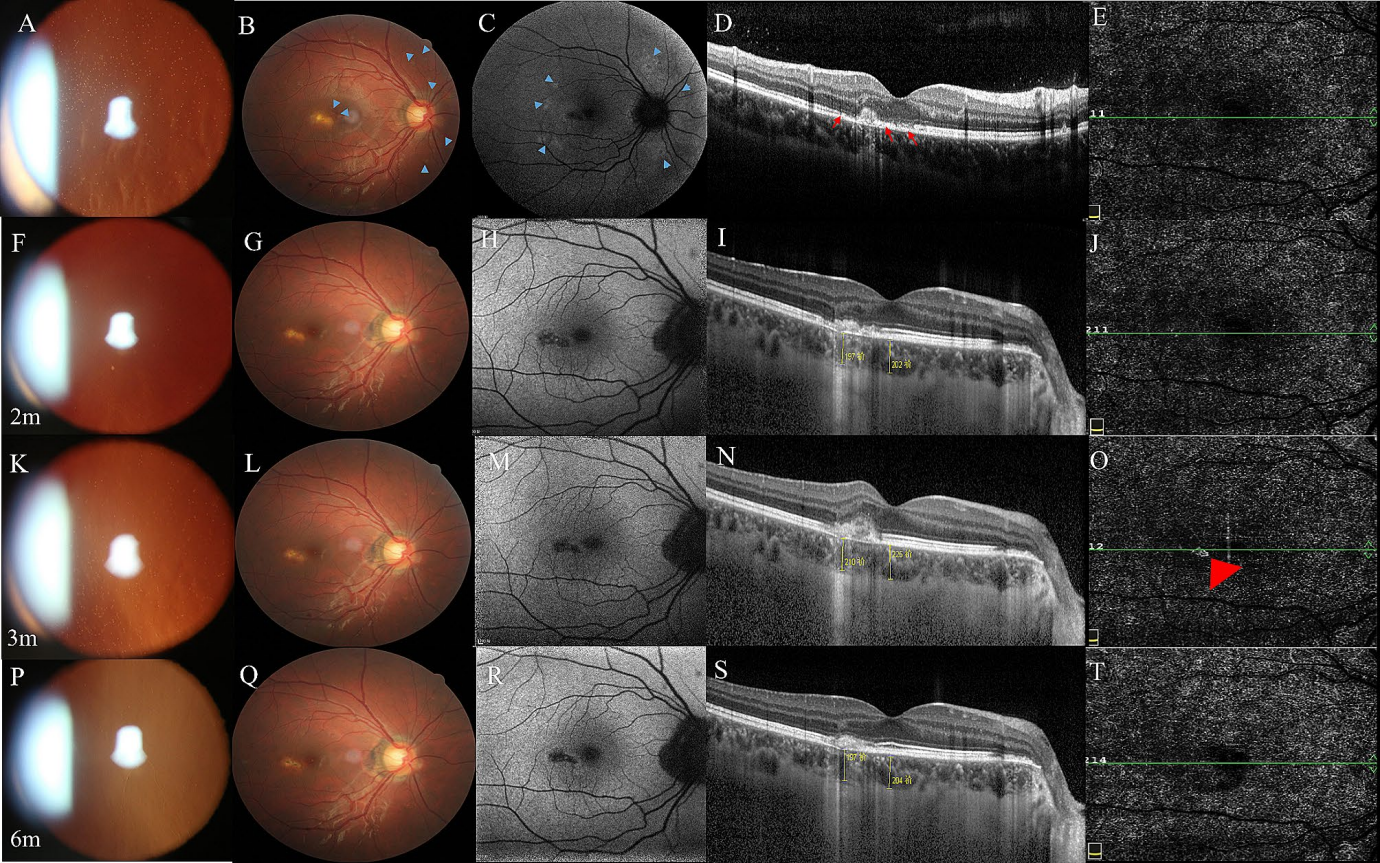
Case 4: First visit (2 weeks after onset) (**A-E**). Two-month follow-up visit (**F-J**), and follow-up visit when a CNV was found (**K-O**). Six-month follow-up visit (**P-T**). **A**: Inflammatory cells in the right vitreous body. **B**: White dots and spots in the macula (blue arrows). **C**: FAF showed diffuse and scattered hyperautofluorescence in the posterior pole. **D**: SHRM and the disruption of RPE-BM-CC in the subtemporal fovea on OCT. **E**: No neovascularization on OCTA. **F**: Obvious reduction of inflammatory cells in the right vitreous body. **G**: The yellowish-white lesion shrank. **H**: FAF showed hypoautofluorescence in the subtemporal fovea. **I**: OCT showed the heterogeneous SHRM shrank. **J**: No neovascularization on OCTA. **K**: Increase of inflammatory cells in the right vitreous cavity. **L**: The lesion in the subtemporal fovea became larger when oral prednisone was reduced to 10 mg/day. **M**: FAF showed hypoautofluorescence in the subtemporal fovea. **N**: Elevation of the SHRM in the subtemporal juxtafovea on OCT. **O**: The neovascularization was visible on OCTA (red arrow). **P**: Significant reduction of inflammatory cells in the right eye. **Q**: The yellowish-white lesion in the right eye shrank with pigmentation. **R**: FAF showed hypoautofluorescence in the subtemporal macula. **S**: The SHRM shrank on OCT. **T**: The neovascularization subsided on OCTA

A 25-year-old female presented with acutely decreased visual acuity and scotoma in her right eye for one week. Her spherical equivalent refractive error was − 2.75 D OD and − 2.50 D OS. Inflammatory cells were noted in the anterior chamber and the vitreous cavity of her right eye, while the left eye was normal (Fig. 6A). CFP showed a yellowish-white lesion in the subtemporal fovea, foveal granularity, and scattered grayish-white dots in the posterior pole (Fig. 6B). FAF showed hyperautofluorescent dots in the posterior pole (Fig. 6C). These manifestations were consistent with the diagnosis of MEWDS. On OCT, the inhomogeneous SHRM in the subtemporal fovea and disruption of EZ were noted (Fig. 6D). No neovascularization was seen on OCTA (Fig. 6E). He was given one retrobulbar TA 20 mg injection and oral prednisone. Two months later, inflammatory cells were reduced in the vitreous cavity (Fig. 6F). The yellowish-white lesion became smaller than before (Fig. 5G). FAF showed hypoautofluorescence in the sub-temporal area of the fovea (Fig. 5H). The SHRM lesion significantly shrank and disruption of RPE-BM-CC occurred on OCT (Fig. 6I). There was no neovascularization found on OCTA (Fig. 6J). When the oral corticosteroid was reduced to 10 mg/day (about three months after the onset of symptoms), the patient complained of severer metamorphopsia with an increase of inflammatory cells in the vitreous cavity (Fig. 6K). CFP of the right eye revealed no obvious change of the juxtafoveal lesion compared to the CFP at the two-month follow-up visit (Fig. 6L). The juxtafoveal lesion showed hypoautofluorescence on FAF (Fig. 6M). The heterogeneous SHRM on OCT elevated higher compared to the two-month follow-up visit imaging (Fig. 6N). Neovascularization could be found on OCTA (Fig. 6O). She was then given an intravitreal anti-vascular endothelial growth factor (anti-VEGF) drug injection and two retrobulbar TA injections. After six months, the inflammatory cells in the anterior chamber and vitreous cavity significantly reduced (Fig. 6P). The yellowish-white lesion became better defined and pigmented (Fig. 6Q). FAF still showed hypoautofluorescence at the lesion’s site (Fig. 6R). OCT showed an obvious shrinkage of the heterogeneous SHRM (Fig. 6S). OCTA showed that the neovascularization had subsided (Fig. 6T).

## Discussion

The typical MEWDS usually presents a singular acute process and self-limiting course, without permanent disruption in the structural anatomy of the posterior fundus or visual function. In recent years, some literature reported cases showing clinical manifestation of a retinal disease or injury before or after the acute stages of MEWDS. Therefore, the occurrence of MEWDS in association with fundus diseases created a problem if there were other forms of MEWDS. In cases with MEWDS-like reactions, all the associated retinal diseases resulted in the disruption of RPE-BM-CC. The underlying mechanisms responsible for the advent of MEWDS-like reactions may be the immune response to the exposure of retinal antigens due to the disruption of RPE-BM-CC. In addition, focal choroidal excavation (FCE) [14–16] and CNV [8, 17–22] have previously been regarded as uncommon complications of MEWDS, significantly impairing the visual function. However, the inflammatory conditions and the disruption of RPE-BM-CC in MFC/PIC cases increased the risks of the development of FCE and CNV. In our study, all of our cases with juxtafoveal yellowish-white MFC/PIC lesions show the MEWDS-like changes around the lesion and disruption of RPE-BM-CC at the lesion’s site. Our cases showed good prognoses and corticosteroid use largely decreased the incidence of FCE and CNV during corticosteroid treatment.

Inflammation involving the outer retina and inner choroid (like white dot syndrome) is a common cause of focal choroidal excavation (FCE). Choroidal excavation is a phenomenon identified on time-domain OCT by Katome et al. [23] and then named by Margolis et al. [24] FCE is associated with congenital choroidal abnormalities and developmental defects, [24] inflammatory diseases [14–16, 21] and choroidal vascular diseases [25]. The pathogenesis of FCE remains unclear. It mostly exhibits a stable state within 1 or 3 years [26]. The inflammatory diseases resulting in FCE include PIC, MFC, Vogt-Koyanagi-Harada syndrome, and MEWDS [27]. After a comprehensive review of published articles, we found that three patients of MEWDS-like reactions with a yellowish-white lesion developed into FCE [14–16] (Table 3). All three reported cases share common features including the increase of sub-lesional choroidal thickness and separation of the RPE from Bruch’s membrane (BrM) by SHRM. The reported patients with mild disruption of BrM were given a small dose of oral corticosteroids for a short term, then the FCE formed about four weeks after onset. Nevertheless, in the reported cases, BrM became continuous at the last follow-up. As time went on, the FCE gradually deepened and then morphologically stabilized. These findings suggest that both the inflammation-induced damage and the self-healing capacity of RPE-BM-CC may be related to the formation of FCE. The small-dose corticosteroids seem not to provide enough anti-inflammatory effects. Under the conditions of FCE associated with inflammatory diseases, the pathogenic progression of FCE was hypothetically elucidated as follows: (1) the inflammation of the outer retina or inner choroid weakens the RPE-BM-CC and then causes fibrous retinochoroidal adhesions at the inflammatory lesion’s sites; (2) the contraction of the fibrous choroidal lesion and intraocular pressure lead to the retinal herniation into the choroid and FCE occurs [28]. In addition, changes of choroidal thickness preceding the development of FCE could also be a potential pathology. In our cases, five patients showed that inflammatory juxtafoveal yellowish-white lesions resolved, and no adhesions formed between photoreceptors and choroid during full-dose corticosteroids treatment. In addition, the disruption of RPE-BM-CC in our patients was mild and resolved within a short duration. Therefore, only a slightly sagged outer retinal layer was observed, instead of FCE.

**Table 3:** Reported cases of MEWDS secondary to MFC/PIC lesions that developed into FCE (the first three) and MEWDS occurring concurrently with CNV (the last 6 cases).i.e. d for day, w for week, m for month, y for year, MTX for methotrexate, PDT for photodynamic therapy, IVR for intravitreal ranibizumab, — for none, FCE for focal choroidal excavation. †, Occurrence time of FCE;‡,Location of CNV /interval between CNV&MEWDS

Gender	Age	Eye	Complaints	History	Refactive state	Choroidal thickness	First vision acuity	Last vision acuity	Anterior chamber cells	Vitreous cells	FCE_†_ or CNV_‡_	Intervention	Follow-up time
Female	37	Right	Blurred vision with scotoma	—	Mild myopia	Increased	20/200	20/25	—	++	4w	Observation	12 m
Female	25	Right	Blurred vision	Prodrome of virus infection	Moderate myopia(after refractive surgery )	Increased	20/50	20/20	—	—	Recurrent FCE with recurrence three times in 2y	Oral glucocorticoid 1.0 mg/kg for 2w	24 m
Female	40	Left	Vision loss	Within 1w after a cold	Not mentioned	Increased	20/32	20/16	—	+	4w	Oral glucocorticoid 15 mg/day for 2w	6 m
Male	16	Left	Vision loss	—	—	Not mentioned	20/40	20/25	—	—	Superonasal side of fovea/1m	Acetazolamide 250 mg for 2w, Bevacizumab 2 mg for 2w, glucocorticoid 100 mg for 3d, 50 mg for 3d. in a total of 2 m	18 m
Female	16	Left	Obvious vision loss	—	Moderate myopia	Increased	20/75	20/75	—	Not mentioned	Temporal side of fovea/concurrent	Observation	20 m
Female	29	Left	Vision loss	Polycystic ovary syndrome	Mild myopia	Increased	20/150	20/60	—	Not mentioned	Fovea/concurrent	Intravitreal anti-VEGF twelve times	18 m
Male	29	Right	Vision loss	—	Moderate myopia	Increased	20/400	20/32	—	Not mentioned	Fovea/concurrent	Observation	3 m
Female	18	Right	Vision loss	—	—	Increased	20/200	20/20	—	Not mentioned	Above fovea/concurrent	Oral glucocorticoid 40 mg/day and reduced gradually for 1 m	1 m
Male	22	Left	Vision loss	Within 2w after a cold	Myopia for 2.5D	Increased	20/200	20/20	Not mentioned	Not mentioned	Superonasal and inferotemporal side of fovea/concurrent	Observation for 7 m and Intravitreal anti-VEGF once	10 m

Choroidal neovascularization (CNV) is a rare complication of primary MEWDS but usually occurs in MFC/PIC cases with MEWDS-like reactions, such as the recurrent MEWDS or MEWDS associated with inflammatory chorioretinal lesions. Notably, in the evolution of recurrent MEWDS, the infiltration and the persistence of inflammation may lead to chorioretinal scar formation, which in turn predisposes to the formation of CNV. In our review of the literature, the occurrence of CNV ranged from four weeks before the onset of MEWDS to 13 years after its onset. There are six reported cases of MEWDS concurring with CNV [8, 17–22]. In these cases, the CNV was mainly located in the juxtafovea, while two cases were around the optic disc (Table 3). One of our cases showed CNV about three months after the occurrence of MEWDS, suggesting a more direct causal relationship between the inflammatory lesion in MEWDS and CNV. An inflammatory lesion with MEWDS-like reactions in juxtafovea were found in 6 cases of published publications [8, 20, 21]. All six cases showed a yellowish-white lesion in the juxtafovea (CNV was subsequently found at the site too), choroidal thickening and subfoveal SHRM. In addition, the typical multifocal deep retinal gray-white spots spread in the posterior pole (Table 3). The features mentioned in the literature are very similar to our 6 patients, while the difference was that 5 of the 6 patients in the literature had MEWDS concurrent with CNV at the first visit. However, our six patients showed no neovascularization at the first visit and only inflammatory lesions in juxtafovea, using a combination of FFA, ICGA, and OCTA examination. After the prompt and full-dose corticosteroid treatment, only one patient developed CNV two months later, with 10 mg/day of prednisone. The reason why the patient had a severer prognosis than the other five patients, was possible that the patient had a severer inflammatory lesion at the site of RPE-BM-CC disruption. In typical cases of MEWDS, ICGA manifested the late-phase hypofluorescence, which might reveal transient intrachoroidal hypoperfusion [2]. Although it is likely that any ischemia at the layer RPE-BM-CC may trigger neovascularization, the mild hypofusion is insufficient to cause permanent structural damage to the overlying RPE and outer retina. However, in cases MFC/PIC lesion, there was a severer choroidal vascular compromise to produce a local ischemic microenvironment in the setting of the rupture of Bruch’s membrane, predisposing to the formation of CNV. We hypothesize that the prompt and full-dose corticosteroid therapy will relieve the inflammation in the choroid and outer retina, which facilitates the rapid repair of inflammatory lesions [26] and reduces the formation of fibrous tissue in the choroid. Thereby the reduced pulling force of fibrous tissue is beneficial to the self-healing of Bruch’s membrane elastic and collagen fibers, thus reducing the formation of CNV. Only one case developed CNV. Given the data of our 6 patients, we hold the opinion that the patients with choroiretinal inflammation and disruption of Bruch’s membrane are more likely to develop into MEWDS-like reations [9]. The focal yellowish-white inflammatory lesion in the juxtafovea may be the precursor of inflammatory CNV, and that timely corticosteroid treatment may reduce the progression of inflammatory lesions transforming into FCE or CNV and protect visual function as much as possible. Vienne-Jumeau et al. also proved patients with PIC and MFC should be treated with corticosteroids to prevent CNV development and decrease CNV recurrences [29]. In our study, enhanced depth imaging-OCT (EDI-OCT) also shows the increase of choroidal thickness in the acute phase, indicating that the changes in choroidal thickness and blood flow also play a role in the occurrence of FCE or CNV [30].

Multifocal lesions with impairment and disruption of RPE-BM-CC are often observed in both PIC and MFC. Previous case reports documented MEWDS-like changes in the setting of PIC and MFC [8, 20, 21]. The MEWDS changes in our study are still evanescent and self-limiting. MMI examinations revealed no differences in MEWDS-related MMI findings between primary MEWDS and MEWDS-like reactions. Meng et al. also held the same opinion that no significant differences in demographic, epidemiological, clinical characteristics, and MMI findings were found between the primary MEWDS and secondary MEWDS, apart from a higher degree of myopia in MEWDS secondary to MFC/PIC than primary MEWDS [11]. However, the occurrence of MFC/PIC lesions with MEWDS-like reactions is still noteworthy. The yellowish-white MFC/PIC lesions in our study are inclined to occur next to the fovea. The persistence of the inflammation infiltration easily results in permanent impairment in the macula. Therefore, corticosteroid treatment is needed to relieve the inflammation. In addition, based on our follow-up outcomes after corticosteroids treatment, the yellowish-white lesions in five cases shrank and became better defined and the EZ in fovea became continuous 13 weeks later. More importantly, the SHRM at the lesion’s site was relieved or disappeared 17 weeks later. Finally, the juxtafoveal yellowish-white lesion became pigmented six months later. The visual acuity of five cases returned to 20/20. The timely and full-dose corticosteroid treatment for cases with yellowish-white lesion shows good promise against severe complications like FCE or CNV. Taken together, the cases with yellowish-white chorioretinal MFC/PIC lesions in the current study gained well-controlled prognoses.

We acknowledge that our study has some limitations. First, considering the retrospective study, the interval of follow-up examinations is variable, so some clinical signs and imaging features may be missed. Second, the initial consultation is at different centers, so the different examination machines and operators may contribute few discrepancies. Third, one patient was lost to follow-up after 6 months. In addition, owing to spatial confined, the paper enumerates four fully described cases. Cases 5 and 6 are similar to Cases 1 and 3, so they are summarized in tables. Finally, although MEWDS with MFC/PIC lesions is a rare situation, the sample size of our cases is still limited.

Despite these limitations, our cases extend the clinical spectrum and first report outcomes of MFC/PIC lesions with MEWDS-like features after corticosteroid treatment. The MEWDS or MEWDS-like reactions are still self-limiting. The isolated yellowish-white juxtafoveal MFC/PIC lesion is an initially inflammatory lesion. The juxtafoveal yellowish-white lesion can progress to type 2 CNV, leading to irreversible vision loss without timely intervention. In patients presenting with active yellowish-white MFC/PIC lesions, regular MMI evaluation for follow-up should be initiated to screen the progression from inflammatory lesions to FCE or CNV, as a possible reference for retina specialists. Retrobulbar injection of TA could be an optional treatment for the yellowish-white lesion, due to its rapid effectiveness locally, within the duration of excluding systematic infectious diseases. In addition, regional application of corticosteroids shortened the usage time of systematic corticosteroids. Moreover, the accurate diagnosis of the variant of white dot syndromes could guide clinical treatment and prognostic evaluation to avoid the occurrence of severe complications.

## Data Availability

All data generated or analysed during this study are included in this published article.
